# Supplementary material for: Threshold-adaptive pruning with multi-key homomorphic encryption for communication-efficient secure federated learning
Source: PLoS One. 2026 May 18;21(5):e0349432. doi: 10.1371/journal.pone.0349432 (PMC13183220; doi:10.1371/journal.pone.0349432)
Supplement: Attachment — Submitted filename: report.pdf [file pone.0349432.s001.pdf]

Referee Report of Manuscript PONE-D-25-23533 entitled “**Threshold-Adaptive Pruning with Multi-Key Homomorphic Encryption for Communication-Efficient Secure Federated Learning**” for the journal *PLOS One*

An efficient federated learning system that combines multi-key homomorphic encryption and adaptive channel pruning is presented in the manuscript. The authors present an Adaptive Pruning Multi-Key Federated Learning (APMKFL) scheme that effectively combines model compression and privacy preservation. The approach is technically sound and addresses both communication efficiency and data security concerns in federated learning environments.

A significant contribution that shows consideration for both theoretical soundness and practical relevance is the use of multi-key BGV homomorphic encryption for safe model aggregation.

Experimental validation on MNIST and CIFAR-10 datasets convincingly supports the proposed method’s effectiveness in reducing communication overhead while maintaining accuracy and privacy.

In conclusion, this work is well-structured and presents insightful results. There are a few things that should be addressed before it may be published. We will go into further detail about these problems:

Related Work: The related literature section is somewhat brief. A few recent studies should be highlighted along with their strengths and weaknesses. For example,

https://doi.org/10.1007/s10586-025-05325-w

Consistency in Capitalization: The placement of figures and tables is consistent throughout the paper. The locations of the corresponding explanations are not aligned properly. For consistency and professionalism, the organization of paper should be standardized throughout the paper.

Proofreading: Minor grammatical errors and typos are present throughout the manuscript. A careful proofreading or professional language editing service is recommended to improve overall readability.

With these revisions, the paper's clarity, depth, and overall impact would be significantly enhanced.
