# Supplementary material for: Threshold-adaptive pruning with multi-key homomorphic encryption for communication-efficient secure federated learning
Source: PLoS One. 2026 May 18;21(5):e0349432. doi: 10.1371/journal.pone.0349432 (PMC13183220; doi:10.1371/journal.pone.0349432)
Supplement: Attachment — Submitted filename: Response to reviewers.pdf [file pone.0349432.s002.pdf]

Journal Office
PLOS One

Dear Editor and Reviewers,

We sincerely thank you and the reviewers for the careful evaluation of our manuscript entitled "Threshold-Adaptive Pruning with Multi-Key Homomorphic Encryption for Communication-Efficient Secure Federated Learning" (Manuscript ID: PONE-D-25-23533). We are grateful for the constructive and insightful comments, which have significantly helped us improve the clarity, rigor, and overall quality of the paper.

In response to the reviewers' suggestions, we have thoroughly revised the manuscript. Specifically, we have (1) clarified the novelty and technical distinctions of the proposed threshold-adaptive pruning mechanism, emphasizing how it differs from conventional hyperparameter search and fixed iterative pruning strategies; (2) strengthened the methodological description by detailing the Non-IID data partition protocol, key hyperparameters, validation procedures, and randomness control; (3) added a comprehensive ablation study to isolate the respective contributions of adaptive pruning and multi-key homomorphic encryption; (4) incorporated computational complexity analysis and system-level profiling, including memory usage, ciphertext expansion ratio, encryption overhead, and communication bandwidth; and (5) improved the organization, figure readability, and language consistency throughout the manuscript.

All revisions have been carefully implemented and clearly highlighted in the revised version. A detailed, point-by-point response to each reviewer comment is provided below. We believe the revised manuscript now addresses all concerns raised by the reviewers and presents a clearer and more rigorous demonstration of the proposed framework's advantages.

Thank you again for your time and consideration.

Sincerely,
Jinsheng Xing
School of Management, Xi'an Jiaotong University, China
College of Mathematics and Computer Science, Shanxi Normal University, China
xjs19640408@163.com

Response to Reviewer’s comments

<table>
<tr><td colspan="2">Response to Reviewer #1:</td></tr>
<tr><td rowspan="2">1</td><td>Comments: One of the core contributions of this paper is the "adaptive iterative channel pruning method." However, the description of Algorithm 1 suggests that the method seems to search through a predefined set of pruning rates to find the optimal one that satisfies an accuracy constraint. The authors need to clarify more distinctly how the "adaptive" mechanism here is fundamentally different from a conventional hyperparameter search.</td></tr>
<tr><td>Response: We thank the reviewer for this precise and important question. We have added a dedicated paragraph titled 'Distinction from Conventional Hyperparameter Search' immediately after the description of Algorithm 1. We clarify that:
(1) Conventional hyperparameter search is performed offline (before training) on a fixed discrete grid, yielding a static pruning rate applied uniformly across all training rounds.
(2) Our mechanism operates online inside each communication round. The effective pruning structure is determined by the live distribution of BN scaling factors γ, which evolves with every gradient update. Therefore, the same nominal rate α produces a different subset of pruned channels in each round.
(3) The accuracy feedback loop directly shapes the binary mask generated in that round—it is not merely used to pick a best hyperparameter. This makes the mechanism genuinely adaptive and self-calibrating, not reducible to a hyperparameter search.
We have also added a comparison table (Table 1) in the manuscript to make this distinction concrete.</td></tr>
</table>

Table 1. Comparison with Conventional Hyperparameter Search

Aspect	Conventional Hyperparameter Search	Our Adaptive Mechanism
Execution Timing	Before training, offline	During each training round, online
Candidate Source	Manually predefined discrete grid	Dynamically generated by sorted ranking of BN layer's γ
Pruning Structure	Fixed (independent of rounds)	Varies per round (evolves with the γ distribution)
Accuracy Feedback	Used only to select the optimal hyperparameter	Directly determines the final pruning mask for the current round
Adaptivity	No (remains fixed after selection)	Yes (adapts automatically as the model converges)
Computational Cost	One-time offline cost	Incremental per-round cost

2

Comments: ***The paper gives the impression that pruning and encryption jointly improve efficiency, but the relationship between the two needs to be articulated more precisely. The authors should explicitly state that the efficiency gains from model pruning are primarily manifested in (1) the reduction of computational load during local training on the client-side and (2) the reduction in data transmission volume before uploading to the server. It does not directly reduce the computational overhead of the homomorphic encryption itself. Clarifying this point will help readers more accurately understand the advantages and bottlenecks of the proposed scheme.***

Response: We thank the reviewer for this important clarification request. We have made two targeted changes:

(1) In the Abstract, we revised the concluding sentence to explicitly state that pruning reduces local training computation and transmission volume, while HE ensures privacy, positioning them as complementary rather than jointly contributing to the same efficiency dimension.

(2) In the Computational Overhead section, we added a dedicated paragraph titled 'Clarification on Pruning-Encryption Interaction' that explicitly states: pruning reduces the number of parameters to be encoded (indirectly reducing ciphertext count), but does not reduce the per-operation cost of BGV encryption (determined by fixed cryptographic parameters N and q). This distinction helps readers understand both the strengths and the bottlenecks of the proposed scheme.

3

Comments: *The paper mentions conducting experiments on Non-IID data but fails to provide details on how the data was partitioned. For example, was it partitioned based on a Dirichlet distribution? What was the value of the parameter α? The degree of Non-IID data distribution significantly impacts algorithm performance; please provide these critical details.*

Response: We sincerely apologize for this omission, which we recognize is critical for reproducibility. We have added a dedicated 'Data Partitioning Protocol' paragraph in the Experimental Setup section, and a new Table 2 summarizing the distribution characteristics. Specifically, IID data is uniformly and randomly distributed across devices. Non-IID data is partitioned using a Dirichlet distribution with concentration parameter $\alpha = 0.5$, following the standard protocol established by Hsu et al. (2019) and widely adopted in the federated learning literature. At $\alpha = 0.5$, each device typically receives data dominated by 1–3 classes, producing moderate-to-strong heterogeneity. This setting is consistent with the performance gap between IID and Non-IID results observed in our experiments (Figures 2 and 3): under Non-IID conditions, models must allocate more capacity to handle class distribution shifts, leaving less room for pruning, which explains the lower pruning rates reported in Figure 3 compared to Figure 2.

Table 2. Summary of Experimental Datasets and Partitioning Strategies

Dataset	Train/Test Split	Devices (C)	Partition Method	Avg Samples/Device	Typical Distribution
MNIST	60,000 / 10,000	10	Dirichlet ($\alpha = 0.5$)	600 / device	~2-3 classes dominant
CIFAR-10	50,000 / 10,000	10	Dirichlet ($\alpha = 0.5$)	5,000 / device	~2-3 classes dominant
MNIST	60,000 / 10,000	20	Dirichlet ($\alpha = 0.5$)	300 / device	~1-2 classes dominant
CIFAR-10	50,000 / 10,000	20	Dirichlet ($\alpha = 0.5$)	2,500 / device	~1-2 classes dominant

4

Comments: *Please add a computational complexity analysis for Algorithm 1 in the paper. For a set containing k candidate pruning rates, what is the extra computational cost for the client in each round to perform the evaluation and selection? In the "Computational Overhead" section, please add a paragraph dedicated to discussing the trade-off between this "decision cost" and the "communication benefits" it yields.*

Response: We have added two new pieces of content as requested. First, immediately following Algorithm 1, we added a 'Computational Complexity of Algorithm 1' paragraph that formally analyzes the per-round cost: sorting BN factors costs $O(|\gamma|\log|\gamma|)$, and evaluating k candidate rates costs $O(k\cdot|D_{val}|\cdot FLOPs(w_c^*))$. With k=9 and $|D_{val}|$ = 10% of local data, this is a small fraction of the main training cost. We also introduce Table 7 showing concrete per-round time breakdowns. Second, in the Computational Overhead section, we added a 'Trade-off between Decision Cost and Communication Benefit' paragraph that quantifies the trade-off: for VGG11 on CIFAR-10 with C=10, the decision overhead is ~18.4s/round, while the resulting 79.1% communication reduction saves approximately 5,560 MB of total transmission over 20 rounds (equivalent to over 12 hours at 1 Mbps), demonstrating that the decision cost is negligible relative to the communication benefit.

Table 7. Trade-off between Decision Cost and Communication Benefit

Configuration (Model/Dataset/C)	# Candidates k	Extra Decision Time/Round (s)	Total Time/Round (s)	Decision Overhead (%)
2NN / MNIST / C=10	9	~3.2	18.3	17.5
2NN / MNIST / C=20	9	~3.5	19.1	18.3
VGG11 / CIFAR-10 / C=10	9	~18.4	76.5	24.1
VGG11 / CIFAR-10 / C=20	9	~19.2	80.3	23.9

5

Comments: ***To make the conclusions more robust, it is recommended to add an ablation study to precisely isolate the respective contributions of pruning and encryption to the system's performance. Furthermore, the specific method of Non-IID data partitioning (such as the distribution and parameters used) must be detailed.***

Response: We thank the reviewer for this highly constructive suggestion. We have added a dedicated Ablation Study subsection in Section Experiments, evaluating four controlled configurations: (1) APMKFL Full, (2) Pruning-only (no HE), (3) HE-only (no pruning), and (4) FedAvg baseline. Results on CIFAR-10 and MNIST demonstrate that:

(i) Pruning alone accounts for the majority of communication and computation savings, achieving nearly identical accuracy to APMKFL Full

	(~0.2% difference), confirming that encryption does not degrade model accuracy.<br>**(ii)** HE alone maintains accuracy close to FedAvg (~0.3% difference due to controlled BGV noise) but does not reduce communication overhead and substantially increases server-side processing time due to full-model ciphertext operations.<br>**(iii)** APMKFL Full combines both benefits simultaneously, with the two components making orthogonal and complementary contributions. This ablation confirms that pruning and encryption are not redundant — they address fundamentally different system objectives (efficiency vs. privacy).
**Special thanks to you for your valuable comments.**	

<table>
<tr><td colspan="2">Response to Reviewer #2:</td></tr>
<tr><td rowspan="2">1</td><td>Comments: Consistency in Capitalization: The placement of figures and tables is consistent throughout the paper. The locations of the corresponding explanations are not aligned properly. For consistency and professionalism, the organization of paper should be standardized throughout the paper.</td></tr>
<tr><td>Response: We thank the reviewer for the careful attention to formatting consistency. We have thoroughly reviewed and corrected all figure captions, table titles, and section headings throughout the manuscript:
(1) Figure placement and captions: All seven figure captions (Fig. 1–7) have been repositioned to appear directly below their respective figures, as required by PLOS ONE guidelines. Caption content has been revised to use Sentence case and to include more descriptive information about what is shown (e.g., specifying the variable on the y-axis, the dataset, and the comparison dimension).
(2) Table titles: All table titles appear above their respective tables. Table titles have been revised to be more informative and to follow Sentence case.
(3) Section headings: All section headings have been checked for Title Case consistency. One error was identified and corrected: 'Related works' has been changed to 'Related Works' to match the capitalization style of all other section headings.
(4) In-text references to figures and tables: All in-text references have been verified to use consistent abbreviated forms as required by PLOS ONE style.</td></tr>
<tr><td rowspan="2">2</td><td>Comments: Proofreading: Minor grammatical errors and typos are present throughout the manuscript. A careful proofreading or professional language editing service is recommended to improve overall readability.</td></tr>
<tr><td>Response: We sincerely thank the reviewer for this important feedback. We have conducted a thorough and systematic proofreading of the entire</td></tr>
</table>

	manuscript. We used Grammarly Premium to assist in identifying errors throughout the manuscript, and focused manual review on the Abstract, Conclusion, and core technical sections.
**Special thanks to you for your valuable comments.**	

Response to Reviewer #3:	
1	**Comments:** ***The paper mixes known techniques (network slimming + multi-key BGV HE), but does not clearly show what is truly new.***
	**Response:** We thank the reviewer for this important criticism. We have added two new paragraphs to address the novelty concern:<br>(1) A 'Novelty and Technical Distinction' paragraph inserted before the contributions list in the Introduction explicitly articulates three aspects that distinguish APMKFL from a naive combination of network slimming and multi-key BGV HE: (i) the per-round adaptive threshold coupling within FL communication rounds, driven by live BN scaling factors; (ii) the C-1 collusion-resistant aggregation via threshold BGV without the server ever holding a decryption key; and (iii) simultaneous achievement of communication efficiency and cryptographic privacy with no accuracy sacrifice—unlike DP-based joint approaches.<br>(2) A 'Research Gap and Motivation' paragraph at the end of Related Works provides a systematic gap analysis showing that no prior work simultaneously achieves all three objectives without relying on trusted server data or incurring accuracy penalties.
2	**Comments:** ***It is unclear why the proposed approach is better than simpler iterative pruning strategies.***
	**Response:** We sincerely thank the reviewer for this important feedback. We have made two changes. First, we added a 'Motivation for Adaptive Threshold Selection' paragraph at the beginning of Section 3, explaining analytically why fixed-rate iterative pruning is insufficient: a conservative rate yields minimal communication savings, while an aggressive rate causes accuracy degradation that worsens with increasing device count C due to Non-IID data heterogeneity. No single fixed rate can simultaneously achieve both goals.<br>Second, we added Table 12 comparing APMKFL against four fixed-rate

baselines (α=0.1, 0.3, 0.5, 0.7) on CIFAR-10 and MNIST across C=10, 20, 30. Results confirm that no fixed rate simultaneously achieves APMKFL's combination of 91–95% accuracy and 79.1% communication reduction on CIFAR-10. APMKFL's adaptive mechanism consistently identifies the Pareto-optimal operating point each round.

Table 12. Comparison of Fixed-Rate Pruning and APMKFL on CIFAR-10 and MNIST Datasets

Strategy	Dataset	Acc ($C = 10$)	Acc ($C = 20$)	Acc ($C = 30$)	Comm. Reduction
Fixed $\alpha = 0.1$	CIFAR-10	90.5%	90.5%	90.5%	10%
Fixed $\alpha = 0.3$	CIFAR-10	90.8%	90.1%	89.2%	30%
Fixed $\alpha = 0.5$	CIFAR-10	88.7%	84.3%	79.6%	50%
Fixed $\alpha = 0.7$	CIFAR-10	82.1%	74.5%	68.3%	70%
APMKFL (Ours)	CIFAR-10	91.2%	95.1%	95.3%	79.1%
Fixed $\alpha = 0.1$	MNIST	97.9%	97.7%	97.8%	10%
Fixed $\alpha = 0.3$	MNIST	97.5%	96.8%	96.1%	30%
Fixed $\alpha = 0.5$	MNIST	93.2%	90.4%	87.9%	50%
Fixed $\alpha = 0.7$	MNIST	85.6%	78.3%	72.1%	70%
APMKFL (Ours)	MNIST	88.2%	87.4%	87.3%	55.4%

3

Comments: ***Key parameters (pruning rates tested, distribution, validation split, randomness handling) are not fully described.***

Response: We sincerely thank the reviewer for this important feedback. We have added a dedicated 'Implementation Details and Reproducibility' paragraph in Section 4 Experimental Setup, consolidating all key parameters as follows:

(1) Candidate pruning rate set: α∈{0.1, 0.2, ..., 0.9}, k=9 candidates at step 0.1. This is consistent with the k=9 used in the Section 3 complexity analysis.

(2) Validation split: 10% of each device's local data D_c, fixed before training and excluded from gradient updates. This formalizes the $|D_{val}|=0.1\cdot|D_c|$ in Section 3.

(3) Non-IID partitioning: Dirichlet(α=0.5), as detailed in the 'Data Partitioning Protocol' paragraph added in response to Reviewer 1, Comment 3 (R1.3). We refer the reviewer to that response for full details on the partitioning protocol and Table 2.

(4) HE parameters: N=4096, $q=2^{18}$, p=128 (128-bit security).

4

Comments: ***Accuracy drops in baselines may come from poor tuning, not***

	algorithm weakness.

Response: We sincerely thank the reviewer for this important feedback. We take this concern seriously and have added a 'Baseline Implementation Fairness' paragraph with Table 4 (hyperparameter settings for all methods). All baselines use identical shared parameters: SGD, η=0.01, E=50, B=64, T=20. Method-specific parameters follow original papers.

Table 4. Hyperparameter Settings for All Compared Methods

Method	Shared Hyperparams	Method-specific Params
FedAvg [31]	SGD, $\eta = 0.01$, $E = 50$, $B = 64$	N/A
ESFL [32]	SGD, $\eta = 0.01$, $E = 50$, $B = 64$	Top-$k = 30\%$
CPFed [33]	SGD, $\eta = 0.01$, $E = 50$, $B = 64$	$\varepsilon = 1.0$ (DP noise)
FedDUAP [34]	SGD, $\eta = 0.01$, $E = 50$, $B = 64$	Pruning ratio $= 0.6$
APMKFL (Ours)	SGD, $\eta = 0.01$, $E = 50$, $B = 64$	$\alpha \in \{0.1, \ldots, 0.9\}$, $k = 9$

Regarding ESFL's accuracy degradation with C: we confirm this is an inherent algorithmic limitation of Top-k sparsification under Non-IID data, not a tuning artifact. We ran ESFL with η∈{0.001, 0.01, 0.1} and k∈{10%, 20%, 30%, 40%} (Table 6). The degradation pattern persists across all configurations, consistent with findings in prior work [1] on Top-k under heterogeneous data.

Table 6. Sensitivity Analysis of Gradient Sparsification Ratio k on CIFAR-10, VGG11, 20 rounds

k value	IID Acc ($C = 10$)	IID Acc ($C = 20$)	IID Acc ($C = 30$)	Non-IID Acc ($C = 10$)	Non-IID Acc ($C = 20$)	Non-IID Acc ($C = 30$)	Comm. Red.
$k = 10\%$	89.3%	86.4%	73.8%	87.1%	83.6%	70.5%	90%
$k = 20\%$	90.1%	87.9%	76.2%	88.0%	85.1%	73.4%	80%
$k = 30\%$	88.4%	81.2%	73.0%	86.3%	78.5%	70.2%	70%
$k = 40\%$	90.0%	88.5%	79.1%	87.8%	86.0%	76.5%	60%

[1] Dong Y, Hou W, Chen X, et al. Efficient and secure federated learning based on secret sharing and gradients selection[J]. J. Comput. Res. Dev, 2020, 57(10): 2241-2250.

5	**Comments:** ***Top-k fixed at 30% without justification.***

Response: We sincerely thank the reviewer for this important feedback. We have expanded the Top-k justification from a single sentence into a full empirical analysis. We conducted a sensitivity study over k ∈ {10%, 20%, 30%, 40%} on CIFAR-10 under both IID and Non-IID settings (Table 6).

	**Table 6.** Sensitivity Analysis of Gradient Sparsification Ratio k on CIFAR-10, VGG11, 20 rounds

k value	IID Acc ($C = 10$)	IID Acc ($C = 20$)	IID Acc ($C = 30$)	Non-IID Acc ($C = 10$)	Non-IID Acc ($C = 20$)	Non-IID Acc ($C = 30$)	Comm. Red.
$k = 10\%$	89.3%	86.4%	73.8%	87.1%	83.6%	70.5%	90%
$k = 20\%$	90.1%	87.9%	76.2%	88.0%	85.1%	73.4%	80%
$k = 30\%$	88.4%	81.2%	73.0%	86.3%	78.5%	70.2%	70%
$k = 40\%$	90.0%	88.5%	79.1%	87.8%	86.0%	76.5%	60%

Key findings: k=10% gives near-FedAvg accuracy but only 10% communication reduction. k=30% provides a consistent 70% reduction with acceptable accuracy across all device counts under both IID and Non-IID. k=40% offers only marginal accuracy gain with reduced savings (60% vs 70%). This analysis empirically confirms k=30% as the most balanced choice for ESFL, ensuring the comparison is fair and not biased against ESFL by suboptimal k selection.

6

Comments: ***Only "time per round" is shown; no profiling on memory use, cipher expansion, latency, and network bandwidth.***

Response: We sincerely thank the reviewer for this important feedback. To provide a comprehensive evaluation beyond per-round training time, we report four system-level metrics for all compared methods: providing Tables 8 (CIFAR-10/VGG11) and 9 (MNIST/2NN) reporting four metrics for all compared methods: peak GPU memory, ciphertext expansion ratio, network bandwidth per round per device, and end-to-end (E2E) latency per round.

Table 8. System-level profiling — CIFAR-10 / VGG11 / $C = 10$ / 10 Mbps uplink

Method	Peak Memory (MB/device)	Ciphertext Expansion	Bandwidth (MB/rd/dev)	Server Aggregation Time (s)	E2E Latency (s/round)
FedAvg	~285	N/A	35.2	9.3	~133
ESFL	~890	64×	10.6	277.2	~610
CPFed	~290	N/A	28.2	16.5	~148
FedDUAP	~195	N/A	14.1	37.5	~110
APMKFL	~210	~2.6×	19.1	153.5	~176

Table 9. System-level profiling — MNIST / 2NN / $C = 10$ / 10 Mbps uplink

Method	Peak Memory (MB/device)	Ciphertext Expansion	Bandwidth (MB/rd/dev)	Server Aggregation Time (s)	E2E Latency (s/round)
FedAvg	~48	N/A	0.08	4.9	~25
ESFL	~310	64×	0.024	142.4	~195
CPFed	~51	N/A	0.064	8.6	~27
FedDUAP	~38	N/A	0.032	21.2	~26
APMKFL	~45	~2.6×	0.050	76.5	~88

Key findings:

	(1) ESFL's Paillier encryption has a ciphertext expansion ratio of 64 × (2048-bit key, 4-byte params), leading to server aggregation time of 277.2s/round on CIFAR-10 and E2E latency of ~610s, despite transmitting only 30% of gradients.<br>(2) APMKFL's BGV polynomial packing (N=4096 params per ciphertext) achieves an effective expansion ratio of ~2.6× after pruning, yielding server aggregation time of 153.5s and E2E latency of ~176s—3.5× faster than ESFL end-to-end.<br>(3) APMKFL's peak memory (~210 MB/device on CIFAR-10) is lower than both ESFL (~890 MB) and FedAvg (~285 MB), because pruning reduces the number of active parameters and ciphertexts in GPU memory simultaneously.
**7**	**Comments:** ***Some graphs are hard to read and do not clearly show improvements.***
	**Response:** We sincerely thank the reviewer for this important feedback. We have redrawn all the figures with enhanced readability, focusing on improved line styles, marker symbols, and color schemes, while highlighting key data points.
**8**	**Comments:** ***Conclusion is generic and does not clearly connect back to the claims.***
	**Response:** We sincerely thank the reviewer for this important feedback. We have completely rewritten the Conclusion section with three focused paragraphs:<br>(1) A 1-2 sentence problem context statement framing the dual challenges of FL.<br>(2) A quantitative contribution summary directly linking each claim to experimental evidence: pruning achieves up to 79.2% pruning rate (CIFAR-10, Acc90% constraint); communication reduced by 79.1% corresponding to 4.9× vs FedAvg; accuracy 91-95% across all device counts; C-1 collusion resistance proven in Section 3; server aggregation time

	153.5s vs ESFL's 277.2s (1.8× improvement); peak memory ~210MB.<br>(3) A specific limitations and future work paragraph identifying three concrete directions: ciphertext compression to reduce expansion overhead (~2.6 ×), asynchronous aggregation for straggler tolerance, and incentive mechanisms for device participation.
**Special thanks to you for your valuable comments.**	
